# Correction: A sibling method for identifying vQTLs

**DOI:** 10.1371/journal.pone.0196881

**Published:** 2018-05-01

**Authors:** Dalton Conley, Rebecca Johnson, Ben Domingue, Christopher Dawes, Jason Boardman, Mark L. Siegal

The sixth author's middle name is missing. The correct name is: Mark L. Siegal. The correct citation is: Conley D, Johnson R, Domingue B, Dawes C, Boardman J, Siegal M L (2018) A sibling method for identifying vQTLs. PLoS ONE 13(4): e0194541. https://doi.org/10.1371/journal.pone.0194541

There is an error in affiliation 5 for author Mark L. Siegal. Affiliation 5 should be: Center for Genomics and Systems Biology, Department of Biology, New York University, New York, NY, United States of America.
