# Supplementary material for: A sibling method for identifying vQTLs
Source: PLoS One. 2018 Apr 4;13(4):e0194541. doi: 10.1371/journal.pone.0194541 (PMC5884517; doi:10.1371/journal.pone.0194541)
Supplement: S1 File — Fig A, Estimated coefficients on SNPs for simulated dependent variable with no effects and confounding between a family-level indicator, genotype, and outcome. The red dashed line represents the true snp level effect (β = 0), while the density curves show the range of estimated β^ for each of the models. We see the fixed effects model correctly centers the β^ near the β = 0, while the other family-level random effects (random intercept) and pooled regression show estimates with significant upward bias in the presence of confounding above ρ = 0.1. However, the random effects has the advantage of smaller sampling variance (more efficient estimator) across all levels of confounding because it pools estimates across families. Fig B, Regional linkage map for FHS genome-wide suggestive SNPs for sibling-pair standard deviation in BMI from 1,000 Genomes, CEU Panel. Maps produced by SNAP ([75]). Fig C, QQ plots associated with Manhattan plots in Fig 1. A) Observed versus expected p-value distributions for analysis of sibling-pair standard deviation in height for FHS generation-three respondents with controls for parental genotype, mean height of sibling pair, sex, and sex difference. B) Same as in (A) except for BMI instead of height. Shaded gray regions depict 95% confidence intervals. Fig D, Power to detect an effect size of R2. The figure contrasts power at three potential sample sizes (defined as the number of sibling pairs in the data)(see Methods): 1) the Framingham Heart Study (FHS) sample used in the present analysis; 2) the Adolescent and Longitudinal Study of Health (AddHealth) sample; and 3) the UK Biobank sample. Likewise, the figure contrasts two potential p-value thresholds: p < 10−5 for the discovery analysis; p < 0.05 for the confirmation analysis. The figure shows that although the sample used in the present analysis (FHS) is not adequately powered to detect realistic effect sizes of R2 < 0.01, newly-released datasets with larger sibling subsamples are adequ [file pone.0194541.s001.pdf]

Supporting Information for: *A Sibling Method for Investigating vQTLs*

Figure A Estimated coefficients on SNPs for simulated dependent variable with *no effects* and confounding between a family-level indicator, genotype, and outcome. The red dashed line represents the true snp level effect ($\beta = 0$), while the density curves show the range of estimated $\hat{\beta}$ for each of the models. We see the fixed effects model correctly centers the $\hat{\beta}$ near the $\beta = 0$, while the other family-level random effects (random intercept) and pooled regression show estimates with significant upward bias in the presence of confounding above $\rho = 0.1$. However, the random effects has the advantage of smaller sampling variance (more efficient estimator) across all levels of confounding because it pools estimates across families.

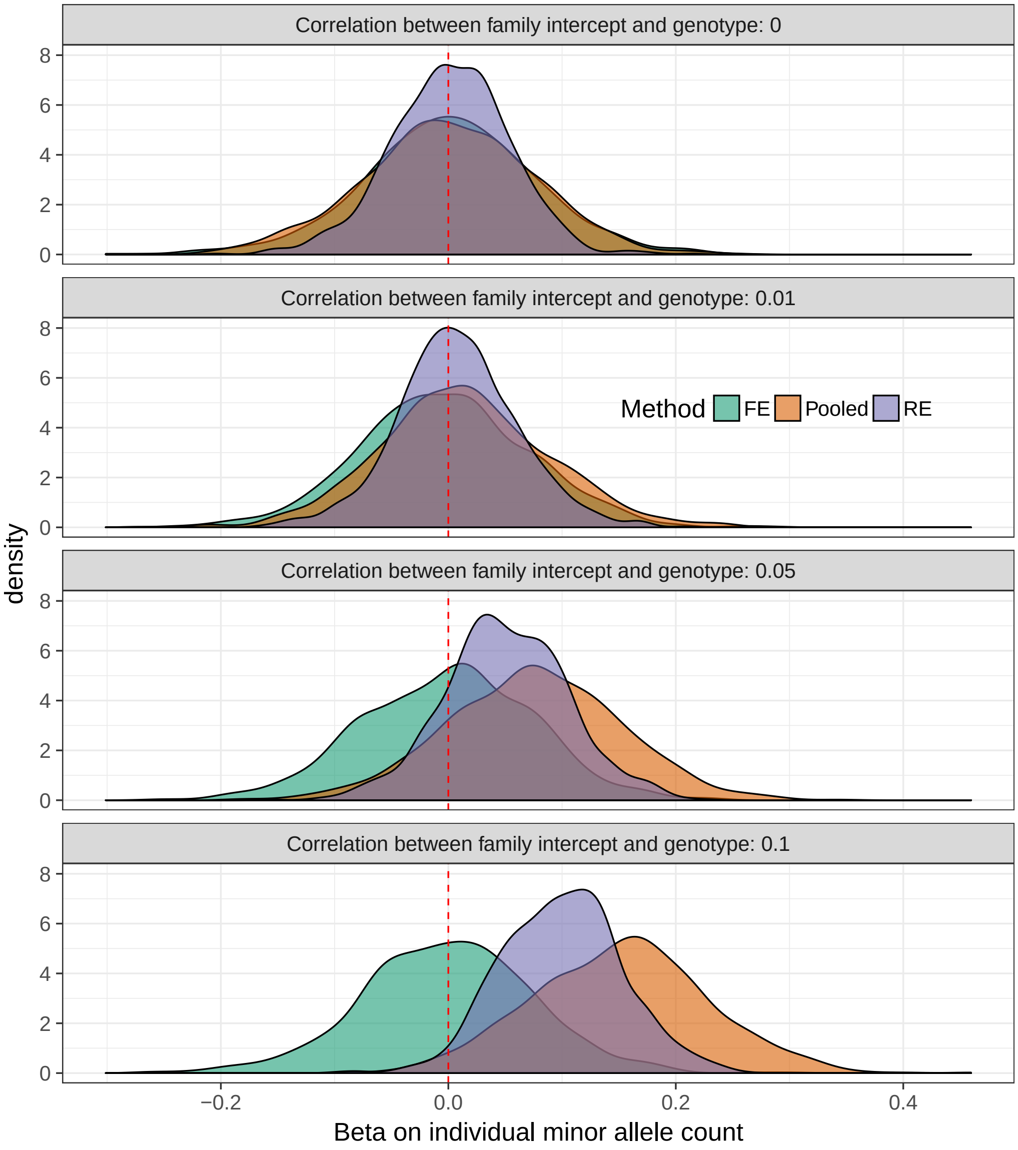

Figure B Regional linkage map for FHS genome-wide suggestive SNPs for sibling-pair standard deviation in BMI from 1,000 Genomes, CEU Panel. Maps produced by SNAP ([47]).

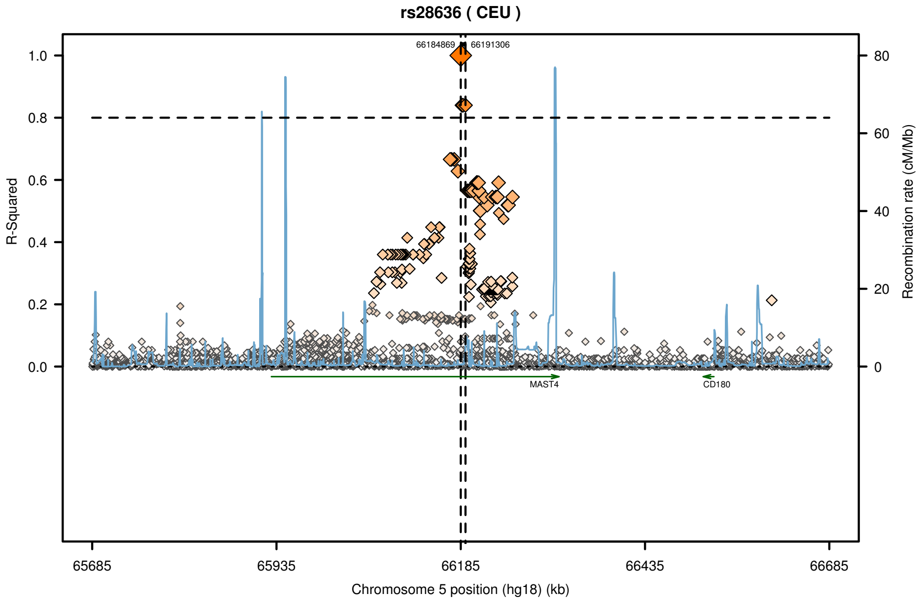

Figure C QQ plots associated with Manhattan plots in Fig. 1 A) Observed versus expected p-value distributions for analysis of sibling-pair standard deviation in height for FHS generation-three respondents with controls for parental genotype, mean height of sibling pair, sex, and sex difference. B) Same as in (A) except for BMI instead of height. Shaded gray regions depict 95% confidence intervals.

A.

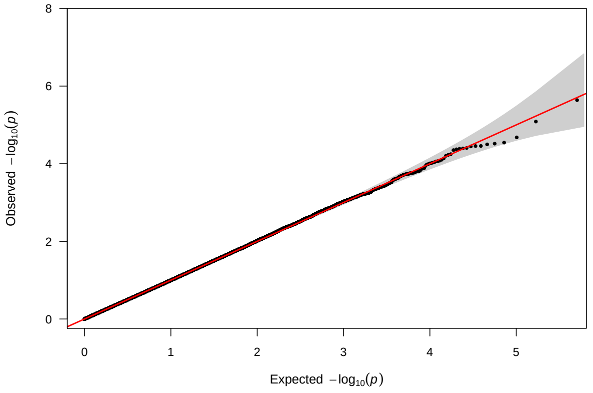

B.

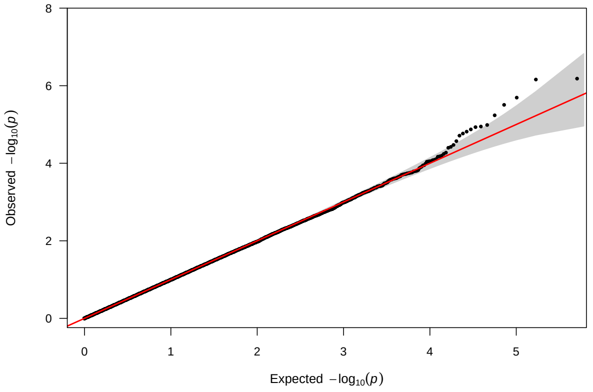

Figure D Power to detect an effect size of R^2 The figure contrasts power at three potential sample sizes (defined as the number of sibling pairs in the data)(see Methods): 1) the Framingham Heart Study (FHS) sample used in the present analysis; 2) the Adolescent and Longitudinal Study of Health (AddHealth) sample; and 3) the UK Biobank sample. Likewise, the figure contrasts two potential p-value thresholds: $p < 10^-5$ for the discovery analysis; $p < 0.05$ for the confirmation analysis. The figure shows that although the sample used in the present analysis (FHS) is not adequately powered to detect realistic effect sizes of $R^2 < 0.01$, newly-released datasets with larger sibling subsamples are adequately powered to detect effects using the method.

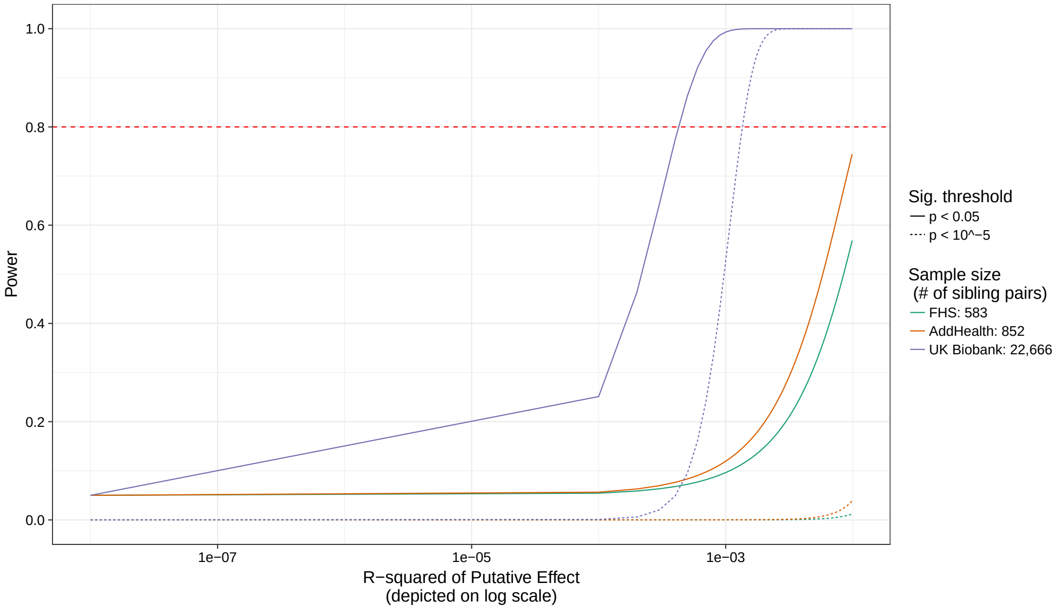

Figure E Regional Association Plot of rs7202116, top hit for variance in BMI found by Yang et al. (2012), on mean level of BMI from GIANT consortium data. Figure produced using LocusZoom ([60]).

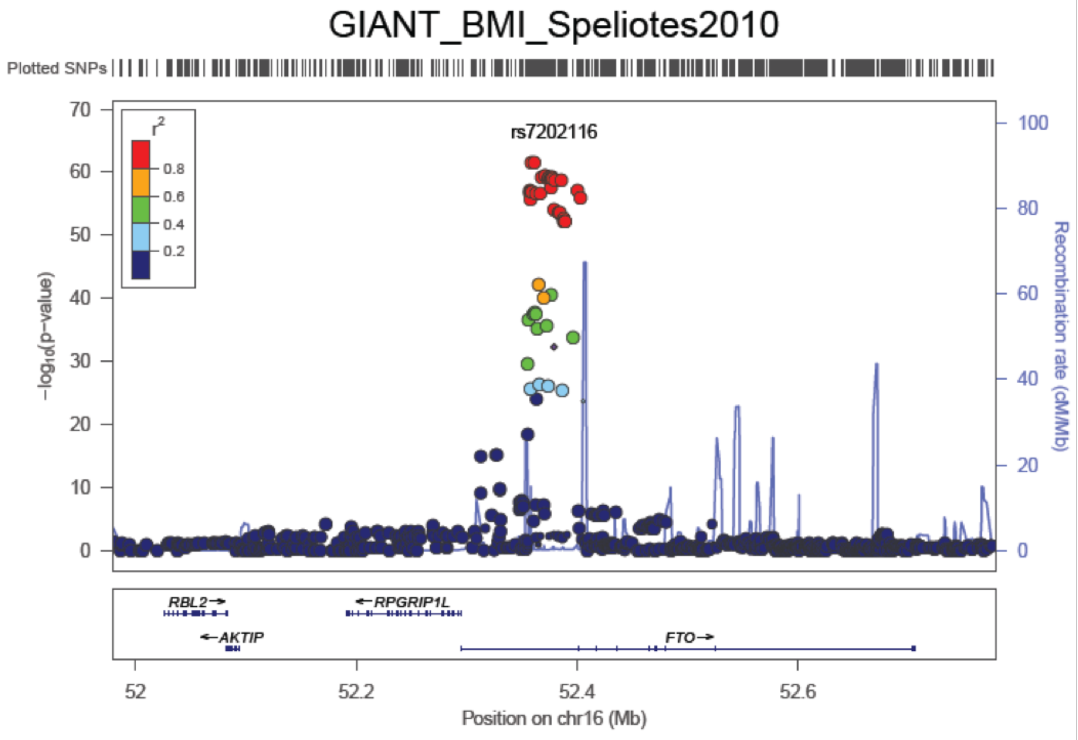

Figure F Regional Association Plot of genome-wide suggestively significant (p<10-5) hits from Fig 1 on mean height from GIANT consortium data. (AD) Plots for the SNPs rs2804263, rs2073302, rs8126205, and rs4834078, respectively, show no markers in the respective regions that approach even genome-wide suggestive significance (p$< 10^{-5}$). Figures produced using LocusZoom ([60]).

A.

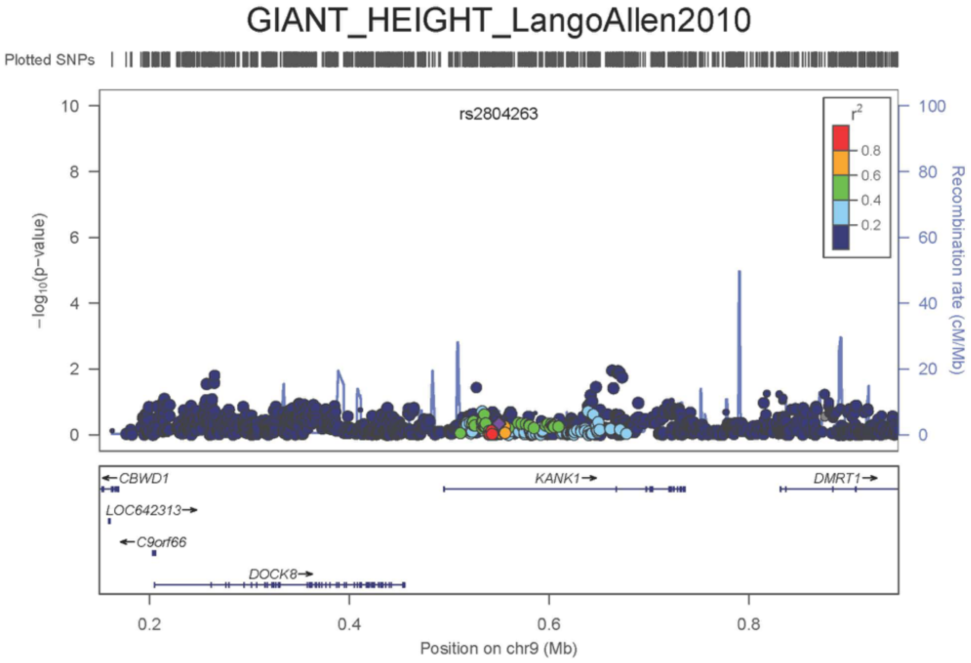

B.

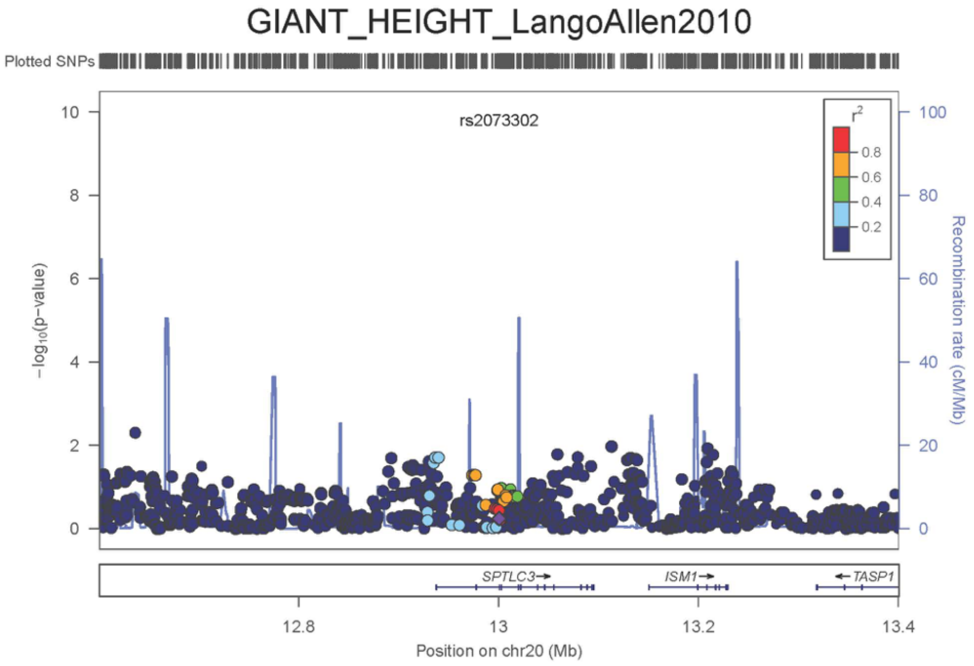
GIANT_HEIGHT_LangoAllen2010
Plotted SNPs
rs2073302
r2
0.8
0.6
0.4
0.2
−log10(p−value)
Recombination rate (cM/Mb)
SPTLC3
ISM1
TASP1
12.8
13
13.2
13.4
Position on chr20 (Mb)

C.

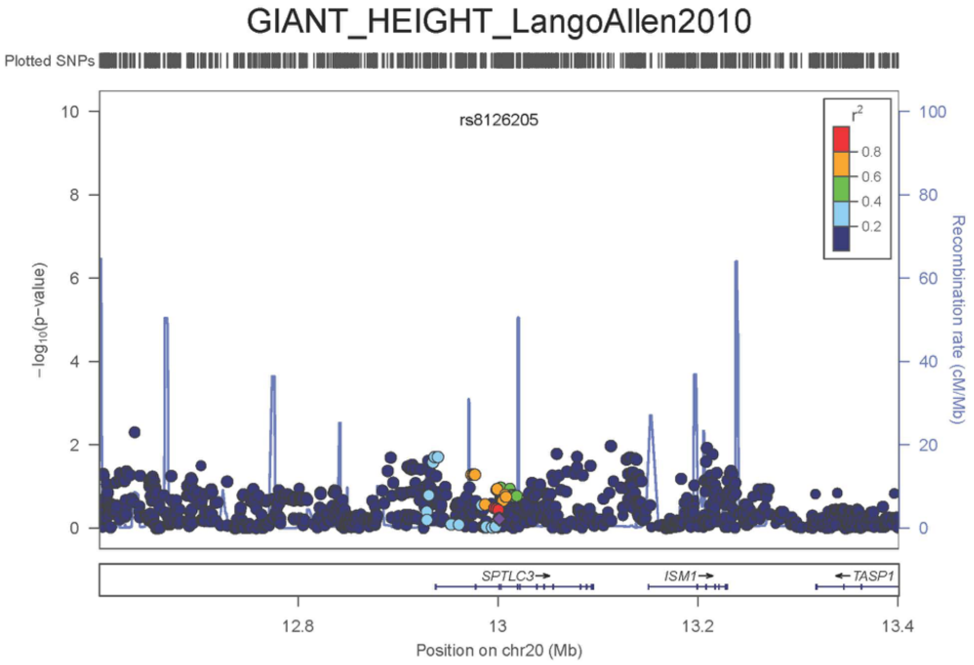
GIANT_HEIGHT_LangoAllen2010
Plotted SNPs
rs8126205
r2
0.8
0.6
0.4
0.2
−log10(p−value)
Recombination rate (cM/Mb)
SPTLC3
ISM1
TASP1
12.8
13
13.2
13.4
Position on chr20 (Mb)

D.

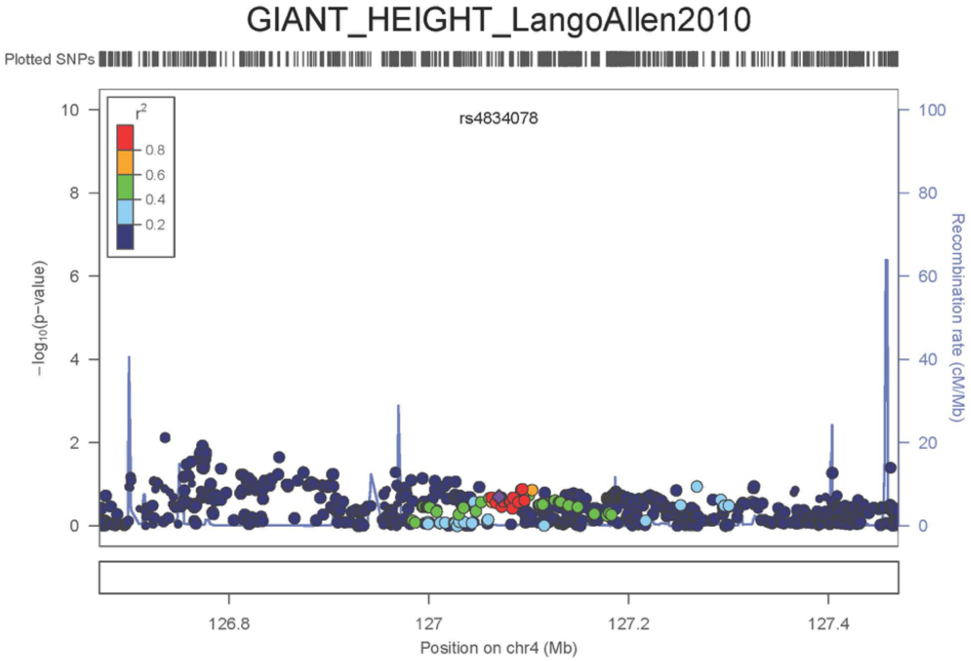
GIANT_HEIGHT_LangoAllen2010
Plotted SNPs
rs4834078
r2
0.8
0.6
0.4
0.2
10
8
6
4
2
0
−log10(p−value)
100
80
60
40
20
0
Recombination rate (cM/Mb)
126.8
127
127.2
127.4
Position on chr4 (Mb)

Figure G Relationship between sibling-pair mean BMI and sibling-pair standard deviation (SD) or coefficient of variation (CV) A) Sibling-pair SD versus mean ($\rho = 0.43$). B) Sibling-pair CV versus mean ($\rho = 0.25$).

A.

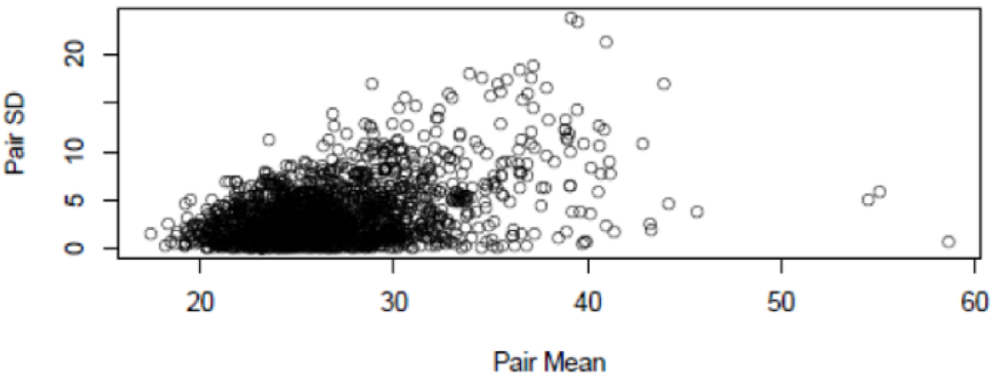

B.

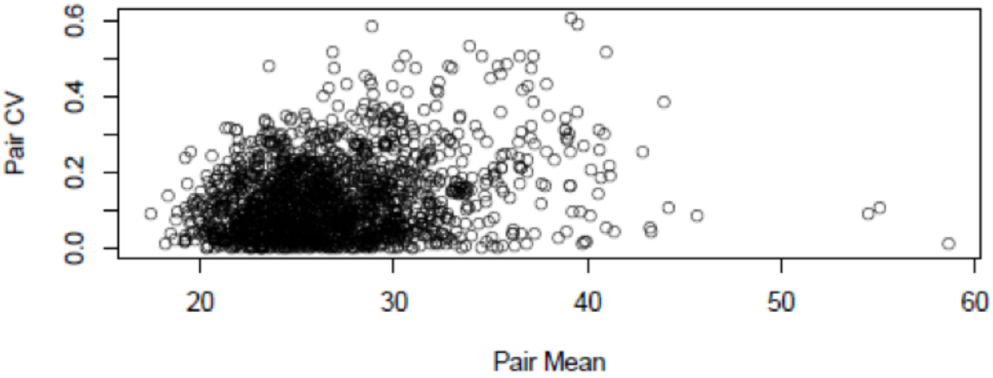

Figure H Manhattan plots for enriched pathway HSA04540 Gap Junction for height variability. A) FHS discovery sample; B) MTFS replication sample.

A.

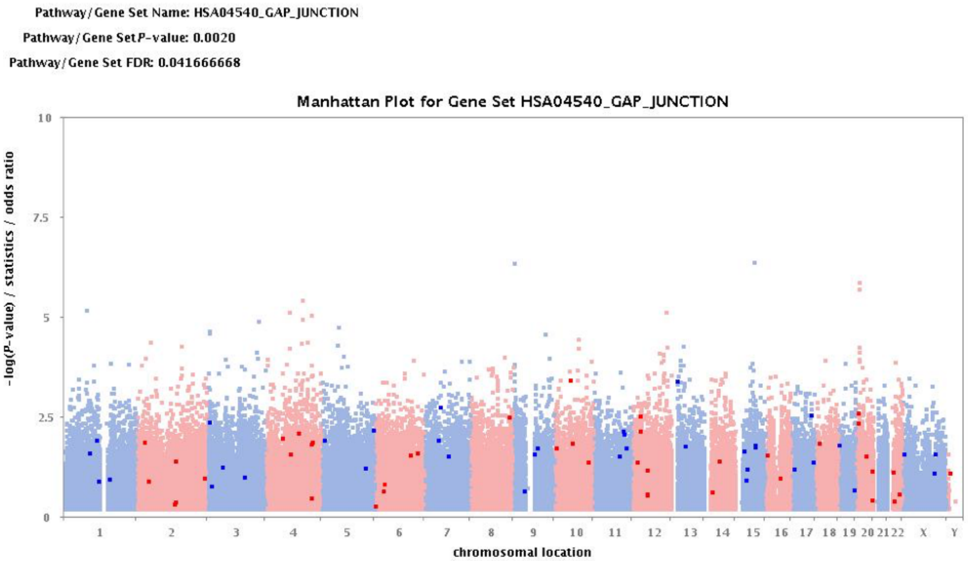

B.

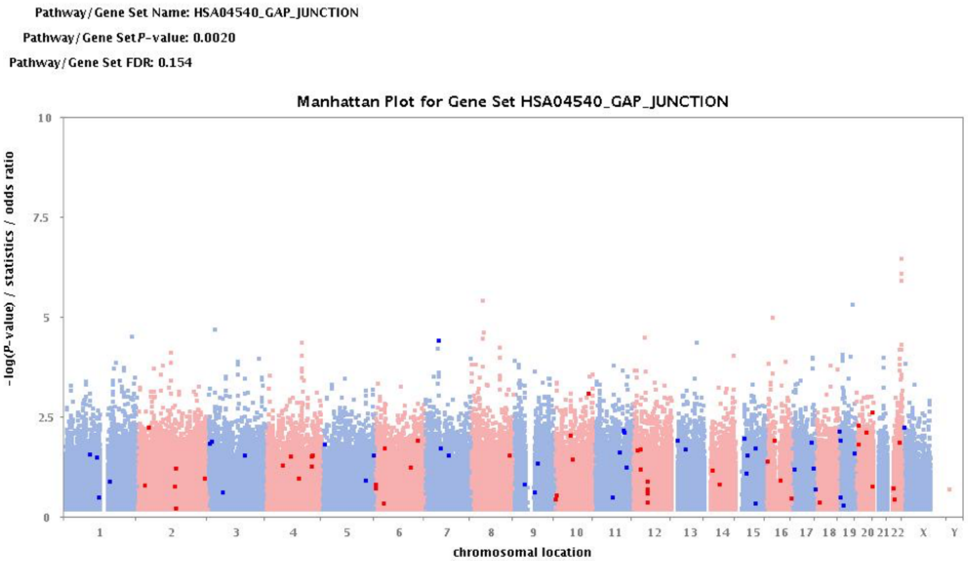

Table A Results of Hausman test comparing $\hat{\beta}_{FE}$ with $\hat{\beta}_{RE}$ across the simulations. The results show that at zero and very small amounts of confounding, the test fails to reject the null hypothesis that $\hat{\beta}_{RE}$ is a consistent estimator, but at higher levels of confounding, the test rejects this null hypothesis.

Correlation level	% of simulations that reject null at $p < 0.05$
0	0.0510
0.01	0.0590
0.05	0.1310
0.1	0.3620

Table B Results of regressing squared Z-score of trait on minor allele count across 1000 replicates with non-demeaned data. The results show an inflated type I error rate for the trait simulated to have either null effects or mean effects but no variance effects in the presence of an unobserved confounder between genotype and outcome (underlined rows).

Simulated DV	Controls for ancestry?	Percent of sims with $p < 0.05$ on minor allele count
No confounding *(*$\rho = 0$*)*		
Neither effects	no	5.10
Neither effects	yes	5.30
Mean effects only	no	5.90
Mean effects only	yes	5.90
Var effects only	no	88.40
Var effects only	yes	87.70
Mean + var effects	no	87.80
Mean + var effects	yes	88.00
Some confounding *(*$\rho = 0.1$*)*		
Neither effects	no	7.10
Neither effects	yes	7.10
Mean effects only	no	8.00
Mean effects only	yes	8.10
Var effects only	no	88.10
Var effects only	yes	87.60
Mean + var effects	no	88.10
Mean + var effects	yes	88.00

Table C Results of regressing squared Z-score of trait on minor allele count across 1000 replicates. Regressions are estimated using the demeaned data. The results show an inflated type I error rate for the trait simulated to have either null effects or mean effects but no variance effects in the presence of an unobserved confounder between genotype and outcome (underlined rows) even after transforming the data.

Simulated DV	Confounding?	Percent of sims with $p < 0.05$ on minor allele count
No confounding ($\rho = 0.0$)		
Neither effects	no	17.1
Mean effects only	no	16.6
Var effects only	no	65.0
Mean + var effects	no	64.7
Some confounding ($\rho = 0.1$)		
Neither effects	yes	16.2
Mean effects only	yes	17.1
Var effects only	yes	66.6
Mean + var effects	yes	66.8

Table D Results of DGLM on non-demeaned (non-transformed) for simulated DV with different types of effects across 1000 replicates. The results show two types of inflated type I error rates. First, when a variant has null effects (neither effects on the mean nor effects on the variance) and there is confounding, the DGLM has an inflated type I error rate, detecting $\beta \neq 0$ in 77.8% of simulations. Second, when a variant has variance effects but no mean effects, the method also has an inflated type I error rate, detecting $\beta \neq 0$ in 11% of cases in the absence of confounding and 70% of cases in the presence of confounding.

Coefficient on minor allele count	% coef $\neq 0$ at $p < 0.05$
**Simulated outcome: neither mean nor var. effects**	
No confounding **(**$\rho = 0$**)**	
β (mean; false positive)	6.6
γ (var; false positive)	0.5
Some confounding **(**$\rho = 0.1$**)**	
β (mean; false positive)	77.8
γ (var; false positive)	0.6
**Simulated outcome: mean effects**	
No confounding **(**$\rho = 0$**)**	
β (mean; true positive)	87.7
γ (var; false positive)	0.8
Some confounding **(**$\rho = 0.1$**)**	
β (mean; true positive)	100.0
γ (var; false positive)	0.7
**Simulated outcome: var effects**	
No confounding **(**$\rho = 0$**)**	
β (mean; false positive)	11.1
γ (var; true positive)	75.3
Some confounding **(**$\rho = 0.1$**)**	
β (mean; false positive)	70.0
γ (var; true positive)	76.7

Table E Results of DGLM on demeaned (non-transformed) for simulated DV with different types of effects across 1000 replicates. The results show an inflated type I error rate (estimate $\beta \neq 0$ despite the presence of allele affects on the variance and not the mean) that is smaller but still present in the demeaned data. The results also show that while demeaning reduces the type I error rate (false detection of mean effects), the transformation leads to type II errors (fails to detect variance effects when these are present).

Coefficient on minor allele count	% coef $\neq 0$ at $p < 0.05$
**Simulated outcome: neither mean nor var. effects**	
No confounding **(**$\rho = 0$**)**	
β (mean; false positive)	17.4
γ (var; false positive)	0
Some confounding **(**$\rho = 0.1$**)**	
β (mean; false positive)	15.6
γ (var; false positive)	0
**Simulated outcome: mean effects**	
No confounding **(**$\rho = 0$**)**	
β (mean; true positive)	82.9
γ (var; false positive)	0
Some confounding **(**$\rho = 0.1$**)**	
β (mean; true positive)	80.7
γ (var; false positive)	0
**Simulated outcome: var effects**	
No confounding **(**$\rho = 0$**)**	
β (mean; false positive)	19.0
γ (var; true positive)	0
Some confounding **(**$\rho = 0.1$**)**	
β (mean; false positive)	20.5
γ (var; true positive)	0

Table F Results of regressing sibling SD of trait on minor allele count across 1000 replicates. The results show the percentage of simulations for which the coefficient on the minor allele count is significant at the $p < 0.05$ level when we regress the sibling standard deviation of the trait on this count and controls. In order for the method to adequately control for Type I error, we want this percentage to be low for the traits simulated to have 1. neither mean nor variance effects or 2. mean effects only. In order for the method to be adequately powered, we want this percentage to be high for the traits simulated to have: 1. variance effects only and 2. both mean and variance effects. For the first goal, the results show that in contrast to the squared Z-score and DGLM, which each, in the presence of an unobserved confounder, display type I error rates of around 20% in detecting variance effects in traits simulated to have mean effects only, the sibling SD method avoids this type of error (underlined rows) both with and without controls for parental genotype. The results also illustrate that the method detects variance effects when the trait either has variance effects only or when the trait exhibits both mean and variance effects. The first half of the table also shows a lower type I error rate than squared Z-score when there is no unobserved confounder.

Simulated DV	Controls for parent genotype?	Percent of sims with $p < 0.05$ on minor allele count
No confounding		
Neither mean nor var effects	No	4.30
Neither mean nor var effects	Yes	6.40
Means effect only	No	4.20
Means effect only	Yes	4.60
Var effects only	No	81.60
Var effects only	Yes	40.40
Mean and var effects	No	82.20
Mean and var effects	Yes	40.70
Some confounding ($\rho = 0.1$)		
Neither mean nor var effects	No	4.80
Neither mean nor var effects	Yes	5.40
Means effect only	No	4.70
Means effect only	Yes	5.60
Var effects only	No	80.80
Var effects only	Yes	44.10
Mean and var effects	No	82.10
Mean and var effects	Yes	43.3

Table G Proxy SNPs and results for replication analysis using Minnesota Twin Family Study data.

FHS SNP	MTFS Proxy	Distance	R^2	D	P-value	N (pairs)
**Height SNPs**						
rs2804263	rs2804279	8976	0.959	1	0.174	1555
rs2073302	N/A	–	–	–	–	
rs8126205	N/A	–	–	–	–	
rs4834078	rs4348108	14075	1	1	0.782	1555
rs4834078	rs11730519	23237	0.959	1	0.89	1555
rs4834078	rs6852484	3263	0.851	1	0.75	1555
**BMI SNPs**						
rs30731	rs28636	510	0.85	1	0.028	1555
rs41508049	rs10261193	1624	0.84	1	0.204	1555

Table H Replicated GWAS hits for other SNPs on MAST4 Results are from the NCBI Phenotype-Genotype Integrator

Trait	SNP	P.Value
Autistic Disorder	29456	0.00000
Autistic Disorder	17197559	0.00003
Autistic Disorder	1864036	0.00003
Autistic Disorder	253234	0.00004
Blood Pressure	10515002	0.00000
Blood Pressure	1366275	0.00001
Body Height	1363935	0.00005
Body Height	1363933	0.00006
Body Height	10500564	0.00006
Body Mass Index	25832	0.00000
Body Weights and Measures	1363935	0.00006
Bronchodilator Agents	146002062	0.00000
Bronchodilator Agents	189845032	0.00000
C-Reactive Protein	10056426	0.00006
C-Reactive Protein	26923	0.00009
C-Reactive Protein	26929	0.00010
Carotid Intimal Medial Thickness 1	1697137	0.00000
Child Development Disorders, Pervasive	2801640	0.00001
Child Development Disorders, Pervasive	2968192	0.00001
Child Development Disorders, Pervasive	2561078	0.00003
Chlorine	6878808	0.00001
Cholesterol	1364020	0.00000
Cholesterol, LDL	1364020	0.00002
Diabetes Mellitus, Type 2	1030231	0.00009
Electrocardiography	10514995	0.00000
Epilepsy	39861	0.00000
Forced Expiratory Volume	189845032	0.00000
gamma-Linolenic Acid	1007500	0.00001
HIV-1	1697137	0.00000
Intercellular Adhesion Molecule-1	7714441	0.00000
Linoleic Acid	17278159	0.00000
Linoleoyl-CoA Desaturase	1007500	0.00001
Lipids	1469419	0.00001
Lung Volume Measurements	146002062	0.00000
Lymphocyte Count	16895456	0.00005
Lymphocyte Count	12055346	0.00008
Personality	10052424	0.00006
Platelet Function Tests	9687339	0.00000
Platelet Function Tests	16895178	0.00000
Platelet Function Tests	2545386	0.00000
Platelet Function Tests	10940079	0.00001
Platelet Function Tests	4700148	0.00001
Platelet Function Tests	3111632	0.00001
Platelet Function Tests	10057708	0.00001
Resistin	253234	0.00001
Resistin	29456	0.00006
Sodium	6878808	0.00002
Triglycerides	1469419	0.00001
Triglycerides	2561078	0.00004

Table I Gene set analysis results using PASCAL The table shows significant gene sets in FHS that replicated in MTFS at different p-value thresholds (MAST4, the location of the replicated SNP, does not appear because although it was $p < 0.01$ in the FHS dataset, it was $p = 0.1$ in the MTFS dataset).

P-value threshold	Gene sets significant in FHS and MTFS
Height	
0.050	CMPK1,FOXE3,FOXD2-AS1,FOXD2,LOC441601,MIR4692, EEF1DP3,SCG5,SPECC1,CCDC144CP,DLGAP1-AS5, C19orf40,NANP,LOC100134868,LOC284801,MIR26A1, PARL,LARP1B,FBXO8,LOC100506548,RPL37,SNORD72, CARD6,STAG3L4,EN2,CNPY1,FAM219A,DNAI1
0.01	EN2
BMI	
0.05	NOL9,TAS1R1,NAV1,MIR5191,DUPD1,MPPED2,TCHP,FBF1, KIRREL2,APLP1,LRFN1,GMFG,SAMD4B,RELB, CLASRP,ZNF296,GEMIN7,PPP1R37,TTC27,GPR55, SENP2,CPEB2,CDC20B,MIR449C,UST,MAD1L1
0.01	CLASRP,ZNF296,GEMIN7

Table J Pathway analysis results using PASCAL The table shows significant pathways in FHS that replicated in MTFS at different p-value thresholds. The pathway that replicated using the i-GSEA4GWAS tool is not among those tested by PASCAL

P-value threshold	Pathways significant in FHS and MTFS
Height	
	None
BMI	
0.05	KEGG_ALPHA_LINOLENIC_ACID_METABOLISM<br>REACTOME_ACYL_CHAIN_REMODELLING_OF_PS<br>BIOCARTA_HER2_PATHWAY

Table K Illustrating correlation between population indicators and family-level intercept across 1000 replicates at four degrees of family-level confounding The results show that at low levels of confounding ($\rho \leq 0.1$), the broad ancestry indicators are correlated with the indicator for family genotype but are too broad to fully capture the confounding. As the confounding increases beyond low levels, the ancestry indicators better capture the confounding.

$cor(X_{ij}, \alpha_j)$	Percent significant β on population indicator
0	0.1520
0.01	0.1790
0.05	0.1630
0.1	0.1750

Table L Relationship between family intercept and observed genotype The table shows that as the degree of between-family confounding increases, there is a stronger relationship between the intercept that shifts levels of a trait up or down between families and the genotype.

$cor(X_{ij}, \alpha_j)$	Mean β_1
0	0
0.01	0.0113
0.05	0.0589
0.1	0.1181
